# Neural Mechanisms Underlying Individual Differences in Control-Averse Behavior

**DOI:** 10.1523/JNEUROSCI.0047-18.2018

**Published:** 2018-05-30

**Authors:** Sarah Rudorf, Katrin Schmelz, Thomas Baumgartner, Roland Wiest, Urs Fischbacher, Daria Knoch

**Affiliations:** ^1^Department of Social Psychology and Social Neuroscience, Institute of Psychology,; ^2^Center for Cognition, Learning and Memory, University of Bern, 3012 Bern, Switzerland,; ^3^Department of Economics, University of Konstanz, 78464 Konstanz, Germany,; ^4^Thurgau Institute of Economics, 8280 Kreuzlingen, Switzerland, and; ^5^Department of Neuroradiology, Inselspital, 3010 Bern, Switzerland

**Keywords:** control aversion, decision making, fMRI, freedom, social cognition

## Abstract

When another person tries to control one's decisions, some people might comply, but many will feel the urge to act against that control. This control aversion can lead to suboptimal decisions and it affects social interactions in many societal domains. To date, however, it has been unclear what drives individual differences in control-averse behavior. Here, we address this issue by measuring brain activity with fMRI while healthy female and male human participants made choices that were either free or controlled by another person, with real consequences to both interaction partners. In addition, we assessed the participants' affects, social cognitions, and motivations via self-reports. Our results indicate that the social cognitions perceived distrust and lack of understanding for the other person play a key role in explaining control aversion at the behavioral level. At the neural level, we find that control-averse behavior can be explained by functional connectivity between the inferior parietal lobule and the dorsolateral prefrontal cortex, brain regions commonly associated with attention reorientation and cognitive control. Further analyses reveal that the individual strength of functional connectivity complements and partially mediates the self-reported social cognitions in explaining individual differences in control-averse behavior. These findings therefore provide valuable contributions to a more comprehensive model of control aversion.

**SIGNIFICANCE STATEMENT** Control aversion is a prevalent phenomenon in our society. When someone tries to control their decisions, many people tend to act against the control. This can lead to suboptimal decisions such as noncompliance to medical treatments or disobeying the law. The degree to which individuals engage in control-averse behavior, however, varies significantly. Understanding the proximal mechanisms that underlie individual differences in control-averse behavior has potential policy implications, for example, when designing policies aimed at increasing compliance with vaccination recommendations, and is therefore a highly relevant research goal. Here, we identify a neural mechanism between parietal and prefrontal brain regions that can explain individual differences in control-averse behavior. This mechanism provides novel insights into control aversion beyond what is accessible through self-reports.

## Introduction

When others try to control our decisions, many of us will feel the urge to counteract and thereby reestablish our valued freedom of choice. This aversive reaction to the exogenous control of one's freedom of choice, or in short control aversion, puts a strain on many societal domains, for example, in the form of patient noncompliance to psychiatric therapy (De las Cuevas et al., 2014), adolescent defiance against parents (Van Petegem et al., 2015), or employees' reduced work performance when faced with a restrictive employer (Falk and Kosfeld, 2006). Critically, the degree to which individuals engage in control-averse behavior varies largely, which has been documented in numerous studies (Falk and Kosfeld, 2006; Ziegelmeyer et al., 2012; Schmelz and Ziegelmeyer, 2015). What drives these individual differences in control-averse behavior, however, has remained an open question.

Previous work has shown that individuals whose decisions are controlled by another person often report thoughts about the other person's motives such as distrust and lack of understanding for the other person's decision to control (Falk and Kosfeld, 2006). For example, when an employer requests a minimum effort from her employee, the employee may perceive this as a signal of distrust in her intrinsic work motivation. A separate line of work has highlighted the motivation to restore one's freedom of choice, termed reactance, as the key player in driving control-averse behavior (Brehm, 1966; Miron and Brehm, 2006). For example, the elimination of a choice option can lead to an increased desire for that option, which is interpreted as an indirect strategy of freedom restoration (Miron and Brehm, 2006). Moreover, reactance is assumed to be accompanied by negative affects such as anger (Dillard and Shen, 2005). Therefore, negative affects and individual tendencies to express one's anger outward might contribute to the display of control-averse behavior. The literature thus delivers several plausible variables that might drive individual control-averse behavior. Much of the support to date, however, comes from *post hoc* self-reports or measures of behavioral intentions in hypothetical scenarios. Here, we use a neurophysiological measure of the decision processes during real restrictions of the subjects' freedom of choice. By doing so, we aimed to identify the proximal mechanisms that give rise to individual differences in control-averse behavior. Specifically, we tested whether activation in and functional connectivity with the brain regions that are differentially activated during the restriction of the freedom of choice can explain individual differences in control-averse behavior. Moreover, we investigated to what degree this neurophysiological measure complements and mediates self-report data in predicting individual control-averse behavior.

**Figure 1.**
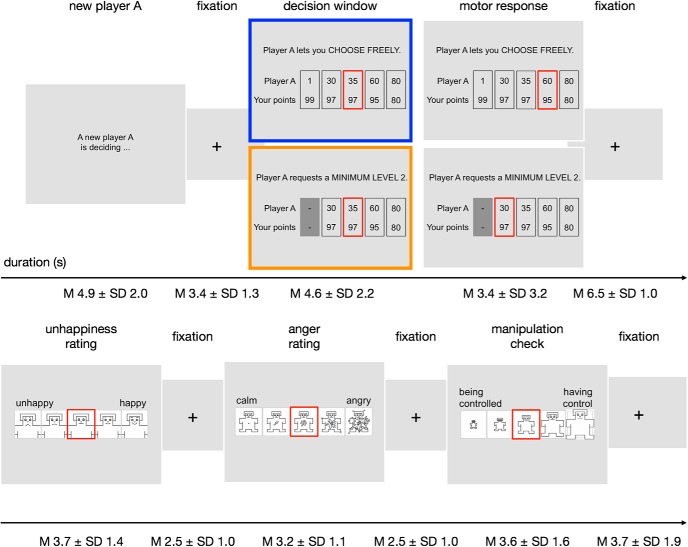
Control aversion task. For every trial, the subject is presented with the decision from a new player A and the available generosity levels. Each generosity level represents an allocation of monetary units between the player A (top value) and the subject (bottom value). In the free condition (blue frame), player A lets the subject choose freely between level one to five (from left to right). In the controlled condition (orange frame), player A requests a minimum of level two and thereby restricts the subject's choice to the levels two to five. The decision window that is highlighted in the figure is defined as the time between the onset of the choice options and the initial movement of the red selection frame. Last, the subject is presented with three pictorial assessment scales, which range from unhappy to happy (left to right), from calm to angry, and from being controlled to having control. The durations of the fixation displays were jittered.

We combined fMRI with a control aversion task (see Fig. 1) in which subjects make decisions that are either free or controlled by another person (Falk and Kosfeld, 2006; Schmelz and Ziegelmeyer, 2015). For each decision, subjects allocate money between themselves and another person by choosing between options that increase in fairness and generosity, called generosity levels. Crucially, the options were designed to establish an intrinsic motivation to choose a high level when subjects can decide freely. When the other person requests a minimum level and thereby tries to control the subject's choice, control-averse behavior is defined as choosing a lower level (Falk and Kosfeld, 2006; Schmelz and Ziegelmeyer, 2015). Therefore, the decrease of average chosen levels when the other person tries to control the subject's decision as opposed to the free decisions serves as a measure of individual control-averse behavior. Critically, the decisions in the task are not hypothetical, but rather have real consequences for both interaction partners and thus share an important quality with control-averse behavior outside the laboratory. This setup allowed us to not only measure control-averse behavior in an ecologically valid fashion, but also to investigate the neural responses during the actual decision-making process. We found that a neural mechanism involving parietal and prefrontal brain regions complements and partially mediates self-reported social cognition in explaining individual differences in control-averse behavior.

## Materials and Methods

### 

#### Participants

We recruited 61 students from the University of Bern for participation in this study. Students of economics, psychology, and social sciences were excluded from participation to reduce the possibility of prior knowledge of the concept of control aversion. All participants were right-handed, nonsmokers, and reported no history of psychological disorders or neurological or cardiovascular diseases. After data acquisition, 10 participants were excluded due to excessive movements during fMRI scan (>5 mm in translation or >5 degrees in rotation), noncompliance to instructions, or technical problems. The remaining 51 participants (23 female; mean age 22 ± 3 SD years) were included in the analysis. All participants received a compensation of CHF 50 (≈50 USD) for participation in the study in addition to the payoff from the control aversion task described in the next section. The study was approved by the Bern Cantonal Ethics Commission and all participants gave informed, written consent.

#### Experimental design

##### Control aversion task.

The control aversion task (see Fig. 1) is designed to confront subjects with real restrictions of their freedom of choice by another person and is based on previous work in behavioral economics (Falk and Kosfeld, 2006; Schmelz and Ziegelmeyer, 2015). The gist of the task is that the subject is asked to allocate money between herself and another person, called player A. However, before the subject makes a decision, player A can decide to let the subject choose freely (free condition) or request a minimum amount of money (controlled condition).

For the purpose of this study, subjects were presented with 16 anonymous other persons' (players A's) decisions from a pilot study in random order. The small number of trials was chosen to increase credibility and reduce possible habituation effects. To ensure equal estimation power of the blood oxygen level-dependent (BOLD) signal across conditions, the players A's decisions were preselected such that the subjects engaged in the same number of trials in the free and in the controlled condition; that is, eight trials per condition. All subjects were informed that the players A's decisions had been prerecorded for logistic reasons and they were asked to decide as if the respective person was present. To remind subjects of this instruction, we presented the line “A new player A is deciding” for a jittered interval of 2.4–8.6 s at the beginning of each trial. Subjects were also informed that their choices had real consequences in the sense that one trial would be randomly selected and paid out to themselves and the corresponding player A. None of the subjects voiced suspicions about the existence of the players A. After a jittered fixation display of 2–6 s, subjects learned whether the player A let them choose freely (free condition) or whether the player A requested a minimum amount of monetary units (MUs) (controlled condition). After a delay of 3 s, subjects made a choice between sets of monetary allocations, called generosity levels, ranging from a selfish (subject: player A, 99:1 MUs) to a more generous, equal allocation (80:80 MUs) (all possible generosity levels are depicted in Fig. 1). Subjects made their choice by moving a red selection frame from a random position to their desired option and pressing an OK button. Response times were not constrained to motivate deliberate decisions; however, subjects were asked to respond as soon as they had come to a decision (response times, mean 5 ± SD 4.3 s). Note that, for the fMRI analysis, we separated the times before and after subjects started to move the selection frame to capture the decision window and the motor responses separately. The durations as used in the fMRI analysis are shown in Figure 1. In the free condition, subjects had the choice between generosity levels one to five (from left to right). In the controlled condition, subjects' choice was restricted to generosity levels two (97:30 MUs) to five. A central feature of the task is that the player A's payoff increases as a concave function of the generosity levels with relatively small and convex costs for the subject. Moreover, the most generous level (level five) also represented the fairest and equal option and the highest sum of payoffs. These features were added to ensure that subjects are intrinsically motivated to choose a high level, which is a prerequisite for control aversion in this task (Schmelz and Ziegelmeyer, 2015). Last, the subject's payoff remains constant for levels two to three. This was done to motivate subjects to choose level three over level two in the free condition, and to provide space for the choice of a lower level in the controlled condition that is independent of economic self-interest. The difference between a subject's mean chosen level in the free condition minus the subject's mean chosen level in the controlled condition served as the measure of the individual level of control-averse behavior.

After another jittered fixation display of 5–8 s, subjects were asked to indicate how they had felt during the decision by rating their unhappiness and anger on 5-point pictorial Self-Assessment Manikin (SAM) scales (Bradley and Lang, 1994), each separated by a jittered fixation display of 1–4 s. The unhappiness scale ranged from 1 = “happy” to 5 = “unhappy” and the anger scale ranged from 1 = “calm” to 5 = “angry.” As a manipulation check, we implemented a third scale, the having control scale, which ranged from 1 = “being controlled” to 5 = “having control.” Finally, a fixation cross was displayed for 1.2–6.4 s before the next trial began.

Before scanning, subjects read the instructions and were quizzed to ensure that they had understood the task and its payoff scheme. Subjects then practiced four simulated trials of the control aversion task outside of the scanner to familiarize themselves with the task timing and the response buttons. Then, subjects completed the scanning task in one continuous session of ∼12 min. At the end of the task, one trial was randomly selected for payoff to the subject and the matched player A. Therefore, all trials were incentive compatible to motivate subjects to decide according to their true preferences. The profits in the selected trial were converted into CHF (with 1 MU = CHF 0.20 ≈ USD 0.20). Based on the task, the subjects received a mean CHF 18.30 ± 1.40 SD and the players A received a mean CHF 11.10 ± 3.80 SD.

##### Ratings of perceived distrust, understanding, freedom restoration, and fairness.

Directly after scanning, we assessed subjects' thoughts during the control aversion task with a list of items. For each item, subjects were asked to rate how strongly the described thought had influenced their decisions on a 7-point Likert scale ranging from 1 = “not at all” to 7 = “absolutely.” Based on the seminal study by Falk and Kosfeld (2006), we assessed subject's perceived distrust and understanding with the items “When player A requests a minimum of generosity, he distrusts me and I dislike that” (“perceived distrust”) and “I understand when player A requests a minimum of generosity” (“understanding”). Based on reactance theory (Brehm, 1966; Miron and Brehm, 2006), we assessed subjects' motivation to restore their freedom of choice in the controlled condition with the item “When player A restricts the generosity levels, I want to use my remaining freedom of choice all the more” (“freedom restoration”). In addition, we asked subjects whether fairness had played a role in their own decisions with the item, “I think that my payoff and player A's payoff should not be too far apart” (“fairness”).

##### Assessment of outward directed anger expression.

To assess subjects' general tendency to direct their anger outward, we asked subjects to fill in the German version of the State-Trait Anger Expression Inventory (STAXI) (Spielberger, 1988; Schwenkmezger et al., 1992). The STAXI is composed of the five subscales state anger, trait anger, inward-directed anger expression, outward-directed anger expression, and controlling one's anger expression. Here, we focused on the subscale for outward-directed anger expression (AO). The AO subscale consists of 8 items that describe ways of expressing one's anger; for example, “I fly off the handle.” Subjects rated these items on a 4-point Likert scale ranging from 1 = “almost never” to 4 = “almost always.” Based on the subjects' ratings, the sum scores were computed. In our sample, the AO subscale had an acceptable internal consistency (Cronbach's α = 0.73). On average, subjects had an AO score of mean 12.24 ± SD 3.02 (range 8–22), which is similar to the norm student sample reported in Schwenkmezger et al. (1992).

#### MRI data acquisition and preprocessing

All MRI data were acquired on a Siemens Trio 3.0 tesla whole-body scanner using a 12-channel head coil. The functional session started off with a localizer scan followed by the control aversion task implemented in E-Prime 3.0 (Psychology Software Tools). The task was projected onto a screen that the subjects viewed through an angled mirror mounted to the head coil. Subjects made their responses on a two-button response box in each hand. While subjects were playing the task, we acquired gradient echo T2*-weighted echoplanar images (EPIs) with BOLD contrast (∼400 volumes per subject, 32 slices per volume, ascending order, field of view 192 × 192 × 110 mm, slice thickness 3 mm, gap 0.45 mm, repetition time 2190 ms, echo time 30 ms, flip angle 90°). Volumes were acquired in axial orientation at a +15° tilt to the anterior commissure–posterior commissure line. After the functional session, T1-weighted 3D-modified driven equilibrium Fourier transformation (MDEFT) images were acquired from each subject (176 slices, field of view 256 × 224 × 176 mm, slice thickness 1 mm, no gap, repetition time 7.92 ms, echo time 2.48 ms, flip angle 16°).

Preprocessing of the functional images was implemented in the MATLAB-based software Statistical Parametric Mapping 12 (SPM12, version r6685; http://www.fil.ion.ucl.ac.uk/spm). Preprocessing included motion correction (realignment to the mean EPI), segmentation of the T1 image into six tissue classifications (gray matter, white matter, CSF, bone, soft tissue, and air tissue), application of this segmentation to the mean EPI, coregistration of all EPIs to the mean EPI using the pullback procedure in the SPM12 deformation tool and normalization of all EPIs to MNI standard space (Montreal Neurological Institute, http://www.bic.mni.mcgill.ca) (Evans et al., 1993). Finally, we smoothed the EPIs with a 4 mm full width at half maximum Gaussian kernel.

#### Analysis aim and structure

The central aim of our analyses was to identify a neurophysiological mechanism that can explain individual differences in control-averse behavior in addition to or beyond self-report data. To this end, our analyses followed a hierarchical structure. First, we identified the best predictor of individual control-averse behavior based on self-report data. Second, we identified a neurophysiological mechanism that predicts individual control-averse behavior. Third, we identified the best combination of predictors based on both self-reported and neural data. Fourth, we tested whether the neural predictor mediates the self-report data in predicting individual control-averse behavior.

#### Behavioral data analyses

All behavioral data were analyzed using the MATLAB Statistics and Machine Learning Toolbox (R2015b; The MathWorks). Because the behavioral data did not follow normal distributions as assessed by Kolmogorov–Smirnov tests, nonparametric tests were applied. Paired samples were compared using the Wilcoxon signed-rank test. Correlations were assessed using Spearman's ρ as well as bisquare robust regressions. For all behavioral analyses, two-tailed *p*-values are reported.

##### Identifying the best predictor of individual control-averse behavior based on self-report data.

We first identified the best predictor of individual control-averse behavior based on self-report data. To this end, we ran a series of generalized linear models using the function fitglm as implemented in the MATLAB Statistics and Machine Learning Toolbox (R2015b; The MathWorks). For each model, the dependent variable was the individual level of control-averse behavior, as measured by the mean chosen level in the free condition minus the mean chosen level in the controlled condition. The self-report variables served as predictors. For conciseness, we report only models with predictors that showed a significant correlation with individual control-averse behavior. To reduce multicollinearity among the predictors, we computed two new variables using principal component analysis as implemented in the MATLAB function pca. The new variable “social cognition” is the first principal component of the normalized ratings of the item “perceived distrust” (coefficient 0.88) and the reversed item “understanding” (coefficient 0.48). The second new variable, “negative affect,” is the first principal component of the normalized mean unhappiness rating (coefficient 0.80) and the normalized mean anger rating in the controlled minus the free condition (coefficient 0.59). As predictors, we used combinations of main effects and interactions of social cognition, negative affect, and the normalized ratings of the item freedom restoration. The most relevant models are illustrated in Figure 4. We compared the models using the Bayesian information criterion (BIC) and *R*^2^ to identify the best model fit. Lower values in BIC and greater values in *R*^2^ indicate better model fits.

#### fMRI data analyses

**Figure 2.**
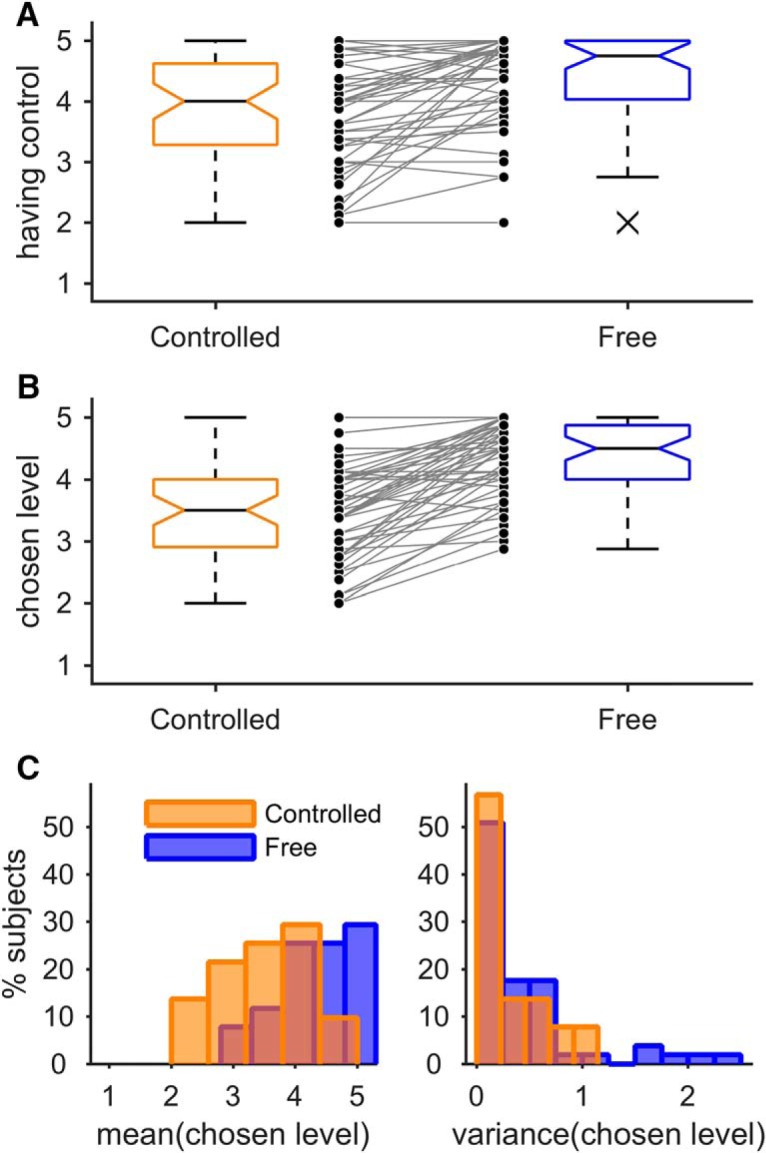
Choice behavior. ***A***, ***B***, boxplots of the ratings of having control and chosen generosity levels, respectively, in the controlled and the free condition. The central mark of each box shows the median, the box edges show the 25th and 75th percentiles, and the whiskers represent the limit beyond which a data point is considered an outlier (denoted as cross). The connected data points in the center show individual subject's means. ***C***, Histograms showing the distribution of subjects' mean and variance of chosen levels in the controlled and the free condition. Data from *n* = 51 subjects are shown.

The statistical analysis of the fMRI data was also performed in SPM12 (version r6685). We modeled each subject's BOLD response with a general linear model (GLM) that was estimated using SPM12's standard hemodynamic response function and a high-pass filter of 128 Hz, as well as correction for intrinsic autocorrelations. SPM12's internal masking threshold for the estimation of the β parameters was set to 0.4 to ensure inclusion of subcortical brain regions. The GLM contained two regressors of interest as boxcar functions: (1) decisions in the controlled condition and (2) decisions in the free condition (each with a duration from the respective onset of the choice options until the first button press, illustrated as “decision window” in Fig. 1). Note that, due to a high consistency in the subjects' choices and therefore in the subjects' and player A's payoff within each condition and subject (see Fig. 2*B*), it was not feasible to additionally control for the subjects' or player A's payoff in the GLM. As nuisance regressors, we also modeled the following: (3) the display of the text “A new player A is deciding …” (duration 2.4–8.6 s); (4) motor response (duration from the first button press until press of the OK button); (5) unhappiness rating (duration = reaction times); (6) anger rating (duration = reaction times); (7) manipulation check, that is, feeling of having control rating (duration = reaction times); and (8) six motion parameters. For every subject, we created contrast images for the two regressors of interest.

At the group level, we used random effects analyses, in which we applied whole-brain correction for multiple comparisons at the cluster level. We calculated the corrected cluster extent (*k*_E_) for each *t* test using Gaussian random-field theory as implemented in SPM12 with a cluster-defining individual voxel threshold of *t* = 2.68 (*p* < 0.005) to achieve an FWE-corrected statistical threshold of *p*_FWE_ < 0.05 (minimum *k*_E_ > 40, range 40–44).

The aim of the fMRI analysis was to identify a neurophysiological mechanism that can predict individual differences in control-averse behavior. Specifically, we investigated whether activations in and interactions with the brain regions that are differentially activated for decisions in the controlled and the free condition correlate with individual control-averse behavior. We did so in three fMRI analysis steps, which will be described in the following sections.

##### Step 1: Localization of brain regions differentially activated for decisions in the controlled and the free condition.

To identify the brain regions that are differentially activated during decisions in the controlled and the free condition, we tested the corresponding contrast images in a paired *t* test at the group level. Because we had no strong anatomical hypotheses, we applied whole-brain corrected analysis. Based on the paired *t* test, we created two masks for all suprathreshold voxels within a 10 mm sphere around the group peak voxel in the right and left inferior parietal lobule (IPL), respectively, at a threshold of *p* < 0.005, uncorrected (peak MNI coordinates for right IPL: 39 −40 40; for left IPL: −42 −40 47, illustrated in Fig. 5). The spheres were applied to isolate the activation in the IPL from more posterior activation. The masks were used to extract and illustrate the mean β estimates as implemented in the MarsBaR toolbox (Brett et al., 2002), as well as for search volumes in the functional connectivity analyses and time course analyses (which are described in step 3 of the fMRI analysis below).

##### Step 2: Covariate analysis of activation differences for decisions in the controlled and the free condition and control-averse behavior.

The second step of the fMRI analysis was to investigate whether individual control-averse behavior could be predicted by activation differences for decisions in the controlled and free condition. To test this, we included the individual level of control-averse behavior as a covariate in the paired *t* test (random effects analysis) using a whole-brain analysis. The individual level of control-averse behavior was computed as the mean chosen level in the free condition minus the mean chosen level in the controlled condition, with the result that increasing values reflect increasing levels of control-averse behavior.

##### Step 3: Covariate analysis of the functional connectivity seeded in the IPL and control-averse behavior.

The third step of the fMRI analysis was to investigate whether individual control-averse behavior could be explained by neural interactions with the brain regions that are differentially active for decisions in the controlled and free condition. For this purpose, we conducted functional connectivity analyses seeded in the right and left IPL as identified in the paired *t* test for decisions in the controlled > free condition. To assess the functional connectivity, we used psychophysiological interaction (PPI) analysis with two psychological factors of interest that were derived from the GLM: (1) decisions in the controlled condition and (2) decisions in the free condition. We extracted single-subject time courses in the right and the left IPL, respectively, as follows: using the search volumes derived from the paired *t* test for decisions in the controlled > free condition at the group level (illustrated in Fig. 6), we identified, for each subject, the peak *Z*-value for the contrast of decisions in the controlled > free condition and extracted the first BOLD signal eigenvariate from a 5 mm sphere around this individual peak. This approach was chosen to account for between-subject variability in the spatial location of the peak activation. The extracted BOLD signal eigenvariate was then deconvolved and multiplied with the two psychological factors of interest to create the PPI terms (controlled PPI, free PPI), which were then convolved with the standard SPM12 hemodynamic response function. Last, for each seed, the two PPI terms, the BOLD signal eigenvariate, and all regressors described in the GLM were entered into a new GLM (GLM-PPI). For all subjects, we created contrast images for the two PPI terms. To identify brain regions that show an increased functional connectivity with the right and left IPL, respectively, we tested the associated contrast images controlled PPI > free PPI in two separate paired *t* tests at the group level (random effects analyses). Finally, to test whether the functional connectivity seeded in the IPL predicts control-averse behavior, we included the individual level of control-averse behavior as a covariate in the paired *t* tests of controlled PPI > free PPI (random effects analyses) using whole-brain analyses.

Based on the covariate analysis, we created two new masks for all suprathreshold voxels in the right and left dorsolateral prefrontal cortex (dlPFC)/middle frontal gyrus, respectively, at a threshold of *p* < 0.005, uncorrected (see Fig. 6, Table 1). These masks were used to extract and illustrate the mean β estimates as implemented in the MarsBaR toolbox (Brett et al., 2002) (Fig. 5) and as search volumes for additional time course analyses (see Fig. 6) as follows.

**Table 1.** Regions in which the connectivity for decisions in the controlled minus the free condition (controlled PPI − free PPI) seeded in the right IPL is positively associated with individual control-averse behavior

Region	Side	MNI coordinates			Cluster size, *k*_E_	Max stat, *t*	*p*_FWE_
		*x*	*y*	*z*			
dlPFC/middle frontal gyrus	R	42	47	22	105	4.88	<0.001
		24	50	5		4.58	
		48	35	29		4.16	
Angular gyrus	L	−33	−55	36	411	4.80	<0.001
		6	−70	50		4.67	
		27	−73	50		4.40	
Precuneus	R	18	−67	29	40	4.80	0.047
		3	−67	29		3.10	
		21	−58	26		3.01	
dlPFC/middle frontal gyrus	L	−45	29	29	41	4.54	0.042
		−39	38	26		3.10	
		−45	35	19		2.95	
IPL	L	−39	−52	57	43	4.22	0.033
		−33	−58	57		3.35	
		−24	−64	60		2.95	

Results from the covariate analysis are shown (sample size, *n* = 51 subjects). Height threshold *t*_(49)_ = 2.68, extent threshold *k*_E_ > 40. All activations survived whole-brain correction for multiple comparisons based on FWE control at the cluster level.

To further examine individual differences in the temporal characteristics of the BOLD signal underlying the decisions in the controlled and free condition in the seed (bilateral IPL) and target regions (bilateral dlPFC/middle frontal gyrus) of the functional connectivity analysis, we performed *post hoc* time course analyses using the search volumes described above. For each subject and each search volume, we identified the peak *Z*-value for the contrast of decisions in the controlled > free condition and extracted the raw event-related BOLD response from a 5 mm sphere around this individual peak, which was identical to the procedure used in the PPI analysis. Event-related BOLD responses were estimated by two finite impulse response models for decisions in the controlled condition and the free condition, respectively, adjusted for nuisance effects of the motion regressors and resampled to time bins of 0.5 s as implemented in the rfxplot toolbox (Gläscher, 2009). We then divided the subjects into groups of not control-averse subjects (with levels of control-averse behavior ≤ 0, *n* = 10) and control-averse subjects (with levels of control-averse behavior > 0, *n* = 41) and plotted the averaged time courses across subjects in each group separately for decisions in the controlled and the free condition (see Fig. 6). Note that the raw event-related BOLD signal is independent of any model assumptions. The time course analyses therefore provide additional insights into the temporal characteristics of the BOLD signal in the target regions. Due to the use of non-independent masks, however, it is important to note that the time course analyses were not used to infer the magnitude of the effect controlled > free condition.

#### Identifying the best combination of predictors of individual control-averse behavior based on self-report and neural data

Building upon the behavioral results and the result of the functional connectivity analysis, we next investigated whether models based on self-report data could be improved by including neural data. To this end, we ran a new series of generalized linear models using the function fitglm as implemented in the MATLAB Statistics and Machine Learning Toolbox (R2015b; The MathWorks). For each model, the dependent variable was the individual level of control-averse behavior, as measured by the mean chosen level in the free condition minus the mean chosen level in the controlled condition.

We compared the best model based on self-report data with models based on the neural data and combinations of neural and self-report data. As a neural predictor, we used the difference between the subjectwise estimate of the connectivity between right IPL and right dlPFC during decisions in the controlled and the free condition (controlled PPI − free PPI). This neural predictor was combined with main effects of and interactions with the predictors social cognition, negative affect, and freedom restoration. The most relevant models are illustrated in Figure 7. Again, we compared the models with regard to the BIC and *R*^2^.

#### Mediation analysis of self-report and neural predictors of individual control-averse behavior

Building upon the result of the model comparisons, we next investigated the association among social cognition, right IPL–dlPFC connectivity, and control-averse behavior. To this end, we performed a mediation analysis using the MATLAB-based mediation toolbox described by Wager et al. (2008) available at: https://github.com/canlab/MediationToolbox. We based the test on three criteria, which are illustrated in the three-variable path model in Figure 8. First, the predictor must be related to the mediating variable (path a). Second, the mediator must be related to the outcome after controlling for the predictor (path b). Third, the mediation effect defined as product of the a and b path coefficients (a*b) must be significant. A significant mediation effect indicates that the mediator significantly reduces and therefore explains the predictor-outcome relationship (difference between path c and c′). If the predictor still explains significant variance in the outcome after controlling for the mediator (path c′), we speak of a partial mediation.

A mediation analysis is conceptually different from a moderation analysis (see model 10 in Fig. 7), which tests whether the level of the moderating variable can predict the strength of the relationship between the predictor and the outcome (Baron and Kenny, 1986; Wager et al., 2008). In other words, a moderator indicates when a predictor-outcome association occurs, whereas a mediator explains how or why such an effect occurs (Baron and Kenny, 1986). We therefore ran the mediation analysis to test whether the right IPL–dlPFC connectivity represents the mechanism through which social cognition affects control-averse behavior.

As the predictor, we used the subject-specific variable social cognition. The mediator was the difference between the subjectwise estimate of the connectivity between right IPL and right dlPFC during decisions in the controlled and the free condition (controlled PPI − free PPI). The outcome was the individual level of control-averse behavior, as measured by the mean chosen level in the free condition minus the mean chosen level in the controlled condition. Statistical significance was assessed using a bootstrap test with 1000 samples.

## Results

### Behavioral results

#### Control-averse behavior and its association with negative affect, perceived distrust, understanding, and freedom restoration

While lying in the fMRI scanner, subjects made choices under two conditions (Fig. 1). In the free condition, subjects could choose freely among five allocation options, called generosity levels, ranging from selfish to more generous and equal monetary allocations between themselves and another person. In the controlled condition, the other person requested a minimum of level two and thereby eliminated the most selfish and unequal option. A manipulation check showed that subjects indeed indicated having more control in the free condition (mean 4.42 ± SD 0.73, median 4.75) than in the controlled condition (mean 3.88 ± SD 0.88, median 4.00; Wilcoxon signed-rank test, two-tailed, *Z* = 4.69, *p* < 0.001, Hodges–Lehmann estimator of differences 0.63, 95% confidence interval (CI): 0.38 to 0.94; Fig. 2*A*).

First, we tested whether the restriction of the freedom of choice had an effect on subjects' generosity as measured by the chosen generosity level. As expected, subjects chose, on average, lower generosity levels in the controlled condition (mean 3.50 ± 0.78 SD, median 3.50) than in the free condition (mean 4.34 ± 0.57 SD, median 4.50; Wilcoxon signed-rank test, two-tailed, *Z* = −5.64, *p* < 0.001, Hodges–Lehmann estimator of differences −1.00, 95% CI: −1.19 to −0.81; Fig. 2*B*). Note that the statistical test was corrected for a bottom effect following the procedure by Falk and Kosfeld (2006). Subjects demonstrated high consistency in their choice preferences: they showed a variance of mean 0.31 ± 0.33 SD, median 0.21, in the controlled condition and a variance of mean 0.33 ± 0.37 SD, median 0.21, in the free condition (Fig. 2*C*). We therefore averaged each subjects' choices within each condition and used the difference between each subject's mean chosen level in the free condition minus the subject's mean chosen level in the controlled condition as the measure of the individual level of control-averse behavior. The individual levels of control-averse behavior varied from −0.25 to 2.13 (mean 0.82 ± 0.64 SD, median 0.88), a variation that stems mostly from the mean chosen levels in the controlled condition rather than the free condition as illustrated in Figure 2, *B* and *C*. In other words, subjects chose similarly high levels in the free condition, whereas choices were more heterogeneous in the controlled condition. For two subjects, the level of control-averse behavior was −0.25, which did not result from systematic choices, but rather from a single outlier choice of a lower level in the free condition. Because these subjects otherwise demonstrated zero difference in their choices between the two conditions, they were treated as not being control averse.

**Figure 3.**
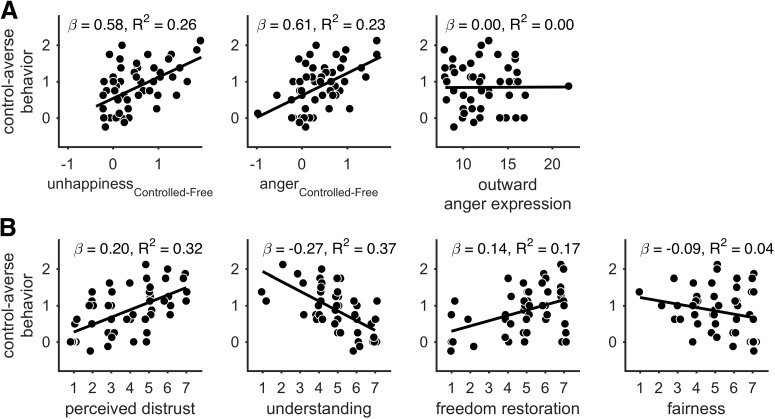
Correlation of control-averse behavior with negative affects, perceived distrust, understanding, freedom restoration, and fairness. ***A***, Mean unhappiness and anger ratings in the controlled minus the free condition and individual tendencies for outward-directed anger expression, respectively, plotted against the individual control-averse behavior, computed as the difference between the mean chosen level in the free minus the controlled condition. ***B***, Individual ratings of perceived distrust, understanding, freedom restoration and fairness plotted against individual control-averse behavior. Observations are jittered along the *x*-axis to reduce overlap for visualization. Regression lines were fitted with bisquare robust regressions. Data from *n* = 51 subjects are shown.

Second, we tested whether subjects' individual control-averse behavior was associated with negative affects (Dillard and Shen, 2005). To capture negative affects, we used trial-by-trial ratings of unhappiness and anger on pictorial 5-point SAM scales (Bradley and Lang, 1994). Indeed, we found a significant association of control-averse behavior with both negative affect ratings: the unhappier (Spearman's ρ = 0.49, *p* < 0.001; robust *R*^2^ = 0.26, *p* < 0.001) and the angrier (Spearman's ρ = 0.46, *p* = 0.001; robust *R*^2^ = 0.23, *p* < 0.001) subjects were in the controlled compared with the free condition, the greater was their individual level of control-averse behavior (Fig. 3*A*). To additionally assess trait anger expression, we used a task-independent anger expression inventory (STAXI; Schwenkmezger et al., 1992). Subjects' general tendency to direct anger expression outward, however, did not correlate significantly with the individual level of control-averse behavior (Spearman's ρ = −0.01, robust *R*^2^ < 0.01, both *p* > 0.9; Fig. 3*A*). Other subscales of the STAXI also showed no significant association with control-averse behavior.

Third, we tested the association between subjects' individual control-averse behavior and their self-reported thoughts as assessed by ratings after scanning. For each rating, subjects were asked to indicate how strongly the described thought had influenced their decision in the control aversion task. Consistent with previous work (Falk and Kosfeld, 2006), we found that subjects demonstrated more control-averse behavior the more they perceived the choice restriction as a signal of distrust by the other person (Spearman's ρ = 0.60, robust *R*^2^ = 0.32, both *p* < 0.001; Fig. 3*B*). In contrast, subjects demonstrated less control-averse behavior the higher they rated understanding the other person's request in the controlled condition (Spearman's ρ = −0.66, robust *R*^2^ = 0.37, both *p* < 0.001). We next tested whether the motivation for freedom restoration had influenced the subjects' decisions. Consistent with reactance theory (Brehm, 1966; Miron and Brehm, 2006), our subjects' self-reported motivation to use their remaining freedom of choice correlated significantly and positively with their level of control-averse behavior (Spearman's ρ = 0.37, *p* = 0.008, robust *R*^2^ = 0.17, *p* = 0.003; Fig. 3*B*). Last, we asked subjects whether fairness had played a role in their decisions; that is, the thought that their own payoff and the other person's payoff should not be too far apart. Interestingly, fairness correlated positively with the average chosen level within both the controlled condition (Spearman's ρ = 0.51, robust *R*^2^ = 0.28, both *p* < 0.001) and the free condition (Spearman's ρ = 0.48, robust *R*^2^ = 0.26, both *p* < 0.001), but was not significantly associated with control-averse behavior (Spearman's ρ = −0.20, *p* = 0.163, robust *R*^2^ = 0.04, *p* = 0.144; Fig. 3*B*).

#### Social cognition is the best self-report predictor of individual control-averse behavior

**Figure 4.**
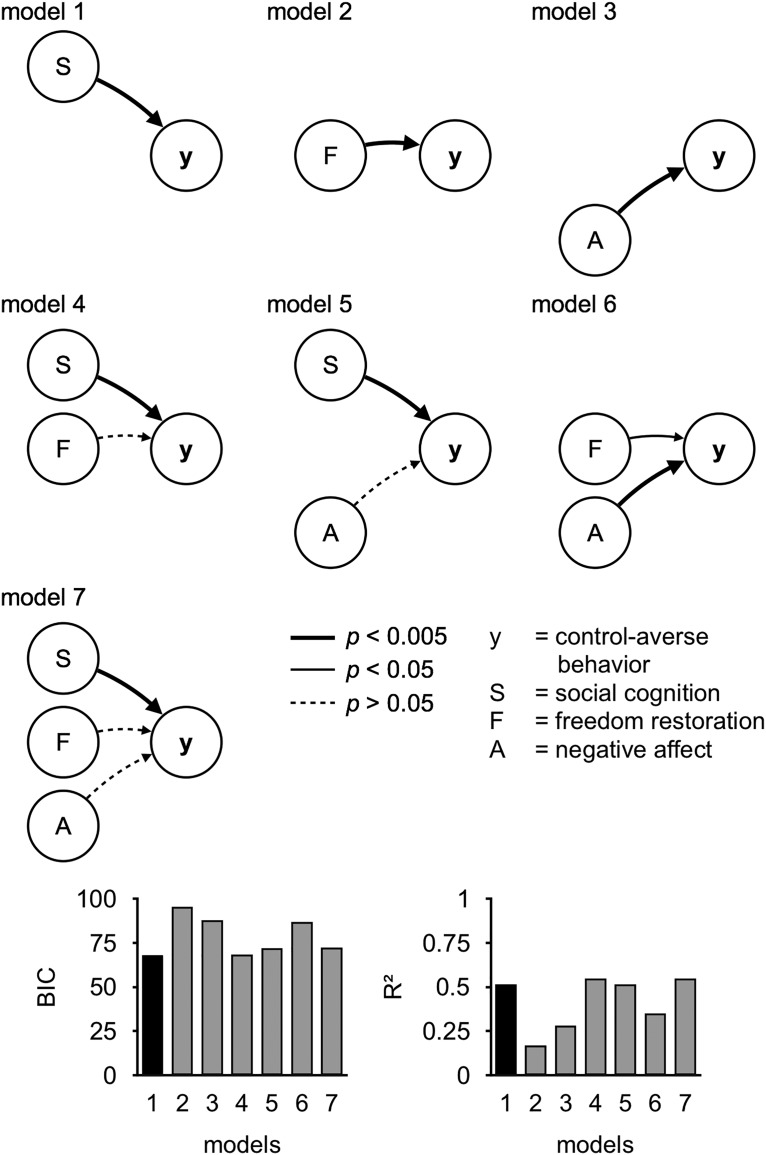
Models based on self-report data. These diagrams show seven models predicting individual control-averse behavior (y), based on self-reports of social cognition (S), freedom restoration (F), and negative affect (A). Arrows indicate main effects. The bar graphs show the BIC and *R*^2^ for each model, with the winning model highlighted in black.

Next, we aimed to identify the best predictor of individual control-averse behavior based on self-report data. To this end, we computed and compared a series of generalized linear models. As predictors, we focused on the self-reported variables that showed a significant correlation with control-averse behavior (Fig. 3). To reduce multicollinearity among the predictors, we applied principal component analyses and computed the new variables social cognition and negative affect. The normalized ratings of the item freedom restoration served as a third predictor. Model comparisons revealed that, based on the self-report data, the following model had the best model fit (Fig. 4, Table 2):

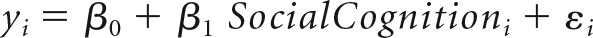
 where *y*_i_ is the level of control-averse behavior for subject *i* and *SocialCognition* is the first principal component of the normalized ratings of the items perceived distrust and the reversed item understanding. This model performed better in predicting individual control-averse behavior than any model that included negative affect or the motivation for freedom restoration either as main effects or interaction terms.

**Table 2.** Model comparison

	Model 1						Model 9					
	β	SE	*t*	*p*	95% CI		β	SE	*t*	*p*	95% CI	
					Lower	Upper					Lower	Upper
Social cognition	1.36	0.19	7.19	<0.001	0.98	1.74	1.06	0.20	5.39	<0.001	0.66	1.45
IPL–dlPFC connectivity							0.92	0.28	3.28	0.002	0.36	1.49
(Intercept)	0.84	0.06	13.51	<0.001	0.72	0.97	0.43	0.14	3.16	0.003	0.16	0.71
BIC	68.1						61.7					
*R*^2^	0.51						0.60					
Observations	51											

The dependent variable was control-averse behavior. Individual differences in control-averse behavior were predicted by social cognition and right IPL–dlPFC connectivity in the controlled minus the free condition (models 1 and 9 in Fig. 7).

### Neuroimaging results

#### Control-averse behavior is predicted by neural interactions between the right IPL and the dlPFC

The aim of the fMRI analysis was to identify a neurophysiological mechanism that can predict control-averse behavior. Specifically, we aimed to test whether neural responses and their interactions could explain individual differences in control-averse behavior. To do this, we ran covariate analyses between the individual control-averse behavior and neural activity in the brain regions that are differentially activated during decisions in the controlled and the free condition, as well as the functional connectivity seeded in these brain regions.

In a first step, the brain regions that are more strongly activated during decisions in the controlled than in the free condition were localized. We estimated a GLM that models the BOLD responses for decisions in the controlled and the free condition, respectively. The respective single-subject contrast images were then compared in a paired *t* test. We found that the right IPL (peak MNI coordinates 39 −40 40, *t* = 3.99, *p*_FWE_ < 0.001, whole-brain FWE corrected at the cluster level), the left IPL (peak MNI coordinates −42 −40 47, *t* = 3.76, *p*_FWE_ = 0.042), clusters in the bilateral superior parietal lobule extending into the occipital cortex (peak MNI coordinates right 15 −73 57, *t* = 4.42, *p*_FWE_ < 0.001; left −21 −64 43, *t* = 4.43, *p*_FWE_ < 0.001), and the right occipital cortex (peak MNI coordinates 39 −79 33, *t* = 4.01, *p*_FWE_ = 0.042) were more strongly activated during decisions in the controlled than in the free condition.

In a second step, we tested whether these activation differences between decisions in the controlled and in the free condition could explain individual differences in control-averse behavior by including the individual level of control-averse behavior as a covariate in the paired *t* test of the contrast images for decisions in the controlled and the free condition. This covariate analysis revealed no significant association between control-averse behavior and the activation differences between decisions in the controlled and the free condition, even at a more liberal statistical threshold of *p* < 0.005, uncorrected.

**Figure 5.**
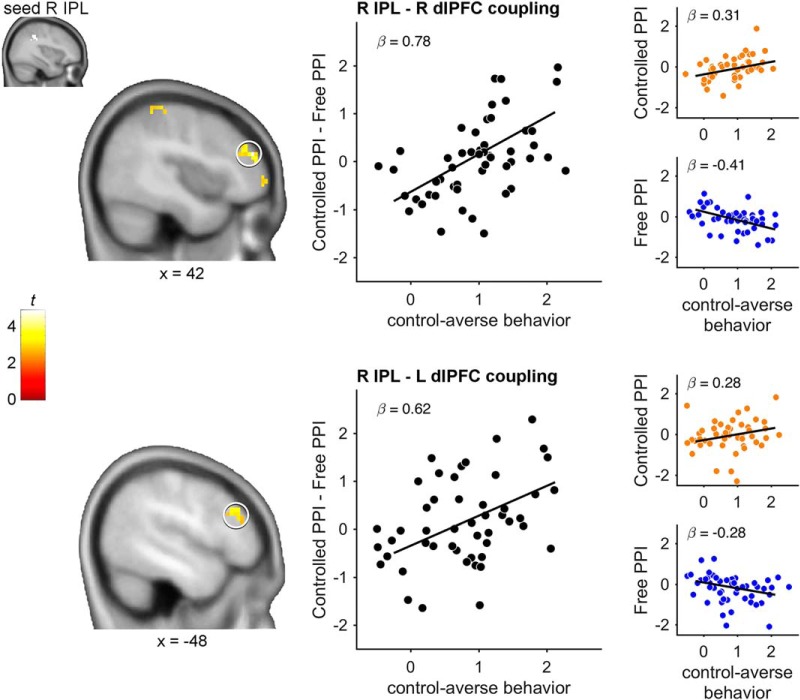
Connectivity between right IPL and dlPFC predicts individual differences in control-averse behavior. The figure illustrates that the functional connectivity during decisions in the controlled as opposed to the free condition (controlled PPI − free PPI) between the right IPL (seed) and regions in the dlPFC/middle frontal gyrus and the posterior parietal cortex increases as a function of individual control-averse behavior. Left, Statistical parametric maps of the covariate analysis color coded for the *t* values as indicated by the color bar, thresholded at *p*_FWE_ < 0.05, and projected on a template brain in MNI space. Right, Graphs showing the individual level of control-averse behavior (*x*-axes) plotted against the single-subject means of the β estimates extracted from the functional clusters in the right and left dlPFC (circled on the left) for the controlled PPI − free PPI effect, the controlled PPI effect, and the free PPI effect seeded in the right IPL (*y*-axes). Observations are jittered along the *x*-axis to reduce overlap for visualization. Regression lines were fitted with bisquare robust regressions. Data from *n* = 51 subjects are shown.

**Figure 6.**
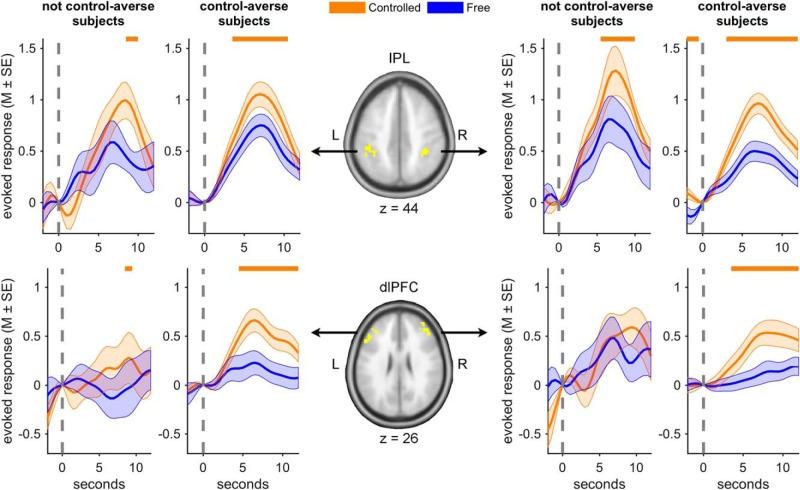
BOLD time courses of decisions in the controlled and free condition. The IPL shows a similar pattern for not control-averse subjects (with levels of control-averse behavior ≤ 0, *n* = 10) and control-averse subjects (with levels of control-averse behavior > 0, *n* = 41), whereas the dlPFC shows a distinct pattern for control-averse subjects. The graphs show averaged time courses of BOLD activation in the bilateral IPL (top row) and the bilateral dlPFC/middle frontal gyrus (bottom row) for decisions in the controlled (orange) and the free condition (blue). The brain maps in the center depict the search volumes used for the time course extractions. The horizontal lines at the top of the graphs indicate time points at which the conditions differ significantly (Wilcoxon signed-rank test, two-tailed, *p* < 0.05). The dashed vertical lines mark the onset of the decision window at which the time courses were mean-corrected. The transparent areas show SEM. Note that these plots were not used to infer the main effect of controlled > free condition.

In a third step, we investigated whether individual differences in control-averse behavior could instead be explained by functional connectivity patterns. As the seed region of the functional connectivity, we focused on the bilateral IPL due to its suggested role in subjective choice restrictions (Filevich et al., 2013) and attention reorientation (Corbetta et al., 2008). Accordingly, the above described peak activation clusters in the bilateral IPL were used as search volumes for individual subjects' seeds for the functional connectivity analyses (Figs. 5, 6). To assess the functional connectivity, we performed two PPI analyses that included separate interaction terms between the right and left IPL BOLD time series, respectively, and regressors indicating decisions in the controlled and the free condition (controlled PPI, free PPI). We searched for brain regions in which functional connectivity with the IPL predicted control-averse behavior by including the individual level of control-averse behavior as a covariate in the paired *t* test of the contrast images for controlled PPI > free PPI. Whereas the covariate analysis seeded in the left IPL revealed no significant results, we found that, for controlled PPI > free PPI, the right IPL showed increased functional coupling with the right dlPFC/middle frontal gyrus (*p*_FWE_ < 0.001), the left angular gyrus (*p*_FWE_ < 0.001), the right precuneus (*p*_FWE_ = 0.047), the left dlPFC (*p*_FWE_ = 0.042), and the left IPL (*p*_FWE_ = 0.033) as a function of control-averse behavior (Fig. 5, Table 1). No significant negative association was observed. Complementary PPI analyses seeded in the superior parietal lobule and the occipital cortex revealed no significant association with control-averse behavior. To determine whether the positive correlation was driven by either one of the conditions, we extracted the mean β estimates across the functional clusters of the bilateral dlPFC for the controlled PPI and the free PPI regressor separately and plotted them against the individual level of control-averse behavior (Fig. 5). This inspection revealed that right IPL–dlPFC connectivity during the decisions increased with control-averse behavior in the controlled condition and decreased with control-averse behavior in the free condition. Therefore, the higher the individual level of control-averse behavior, the greater the change in right IPL–dlPFC connectivity during decisions in the controlled compared with the free condition. In addition, time course analyses showed that activation in the bilateral IPL increases immediately after the onset of the choice options, regardless of individual control-averse behavior (Fig. 6). In contrast, activation in the bilateral dlPFC synchronizes with activation in the IPL only for control-averse subjects and only during decisions in the controlled condition.

#### Connectivity between right IPL and dlPFC complements self-reported social cognition in predicting individual control-averse behavior

**Figure 7.**
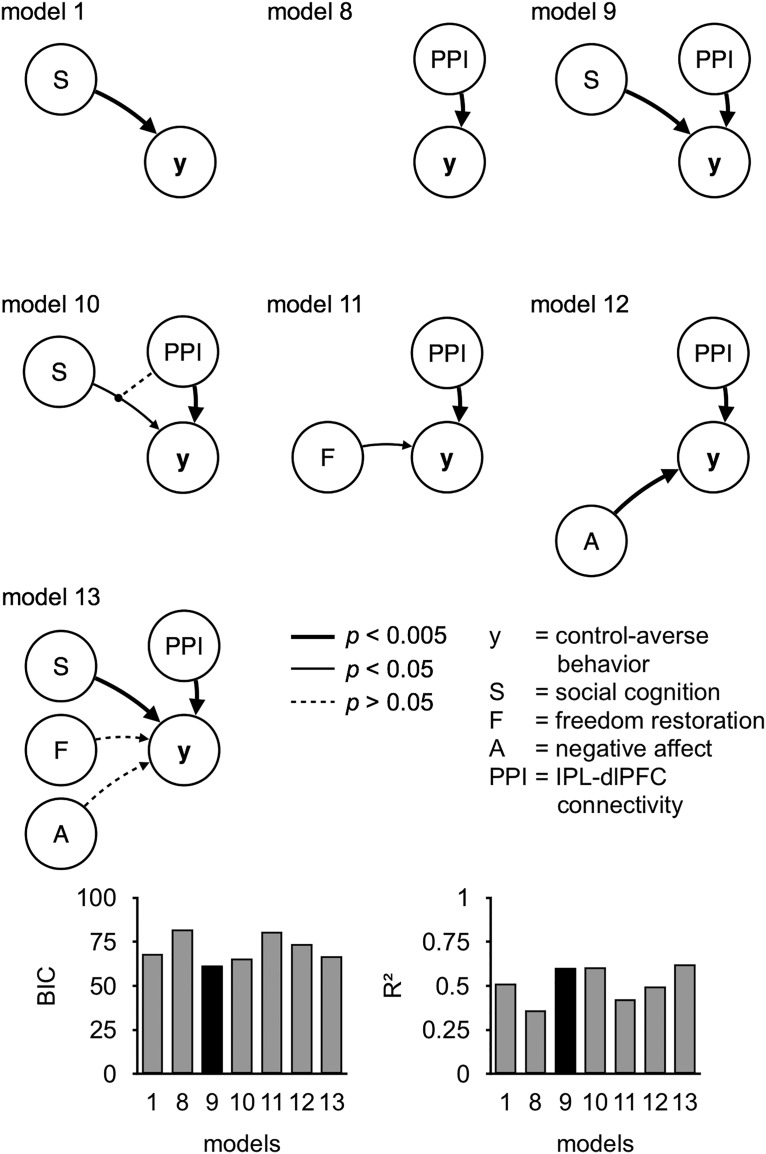
Models based on self-report and neural data. These diagrams show seven models predicting individual control-averse behavior (y), based on self-reports of social cognition (S), freedom restoration (F), negative affect (A), and subjectwise estimates of right IPL–dlPFC connectivity in the controlled minus the free condition (PPI). Arrows indicate main effects and the line with a circular endpoint in model 10 indicates an interaction effect. The bar graphs show the BIC and *R*^2^ for each model, with the winning model highlighted in black.

Next, we aimed to identify the best combination of predictors of control-averse behavior based on both self-report and neural data. Specifically, we tested whether the functional connectivity with the IPL complements or exceeds the self-reports in predicting control-averse behavior. To this end, we computed a set of new generalized linear models that included the neural data. As the neural predictor, PPI, we used the subjectwise β estimate of the controlled PPI minus the free PPI regressor between the right IPL and the right dlPFC. We focused on the connectivity of the right IPL with the dlPFC because of their frequent coactivation during attention reorientation (Corbetta et al., 2008) and context-dependent decision making (Daw et al., 2006; Boorman et al., 2009; Rudorf and Hare, 2014). This neural predictor was combined with main effects of and interactions with the predictors social cognition, negative affect, and freedom restoration. Model comparisons revealed that a model that combined main effects of social cognition and PPI had the best overall model fit (model 9; Fig. 7, Table 2):

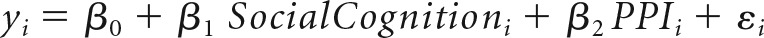
 This model performed better than any combination of the neural predictor with any other predictors based on self-report data (Fig. 7). Moreover, it performed slightly better than a model including the interaction of social cognition and PPI (BIC = 65, *R*^2^ = 0.60, model 10 in Fig. 7), which revealed no significant interaction and therefore no moderation effect (β = −0.40, *t*_(49)_ = −0.38, *p* = 0.702, 95% CI: −2.52 to 1.71). When we added the other self-report predictors (model 13 in Fig. 7), the main effects of social cognition and PPI remained robust, whereas the other predictors showed no significant effect. Accordingly, the increase of connectivity between the right IPL and right dlPFC in the controlled compared with the free condition explains variance in individual control-averse behavior that exceeds model predictions based on self-report data.

#### Connectivity between right IPL and dlPFC partially mediates the association of social cognition with control-averse behavior

**Figure 8.**
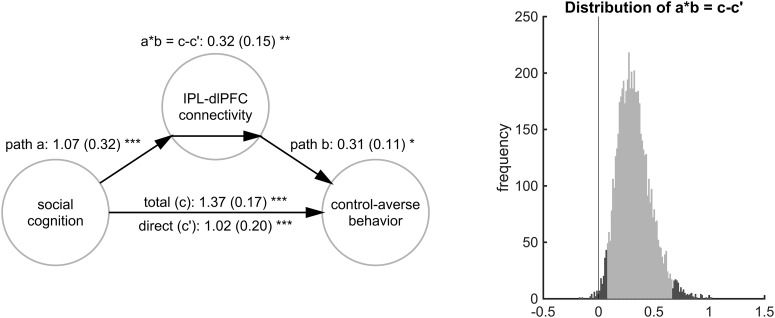
Results of the mediation analysis testing the relationship among social cognition, right IPL–dlPFC connectivity, and control-averse behavior. Left, Model showing the path coefficients with SEM in parentheses, significant at *p < 0.01, **p < 0.005, ***p < 0.001. Right, Histogram of the bootstrapped distribution of the mediation effect (a*b = c–c′). The lighter gray portion of the histogram denotes the 95% CI for the effect. Data from *n* = 51 subjects were included in this analysis.

After having identified social cognition and the right IPL–dlPFC connectivity as the best predictors of individual control-averse behavior, we investigated whether the connectivity might reflect the mechanism through which these social cognitions affect control-averse behavior and therefore capture joint variance. To investigate this, we ran a mediation analysis using a three-variable path model (Fig. 8; Baron and Kenny, 1986; Wager et al., 2008) in which the predictor was social cognition, the mediator was the subjectwise β estimate of the controlled PPI minus the free PPI regressor between the right IPL and the right dlPFC, and the outcome was the individual control-averse behavior. Following convention (Baron and Kenny, 1986), we considered the mediation to be significant if three conditions were met: the predictor must be related to the mediator (path a), the mediator must be related to the outcome after controlling for the predictor (path b), and the mediation effect, that is, the product of the a and b path coefficients (a*b = c–c′), must be significant. The mediation analysis revealed that the relationship between social cognition and control-averse behavior is partially mediated by the connectivity between right IPL and right dlPFC; that is, the mediator significantly reduces the association between predictor and outcome (total effect, path c), but the predictor still explains significant variance of the outcome (direct effect, path c′; Fig. 8). In other words, the right IPL–dlPFC connectivity explains a significant part of the relationship between social cognition and control-averse behavior, but the predictor and mediator each also explain independent variance.

## Discussion

People value their freedom of choice highly. Interestingly, though, if another person tries to restrict one's choice, some people will comply, whereas others will act against the restriction. These individual differences in control-averse behavior have been well documented, but their driving factors have remained a puzzle. Previous work has suggested several potential predictors of control-averse behavior based on self-reports. To date, however, we know very little about the mechanisms that underlie control-averse behavior at the neural level. Here, we identify a neural mechanism that complements and exceeds self-reported social cognitions, affects, and motivations in explaining individual differences in control-averse behavior.

We combined fMRI with a control aversion task (Falk and Kosfeld, 2006; Schmelz and Ziegelmeyer, 2015) in which subjects' freedom of choice is controlled by another person and subjects' subsequent monetary allocation to that person serves as a measure of control-averse behavior. Specifically, we aimed to identify neural mechanisms that could explain individual differences in control-averse behavior. Our results both replicate prior behavioral studies and provide novel insights into the neurobiological basis of control-averse behavior. We replicated that control of one's freedom of choice by another person reduces the willingness to allocate money to that person (Falk and Kosfeld, 2006; Schmelz and Ziegelmeyer, 2015). This effect was augmented in subjects who had little understanding for the other person's behavior or who perceived the restriction of their freedom of choice as a signal of distrust in their intrinsic motivation to choose a generous and fair allocation (Falk and Kosfeld, 2006). We also found that control-averse behavior was accompanied by negative affects (Dillard and Shen, 2005) and the motivation to restore one's freedom of choice (Brehm, 1966; Miron and Brehm, 2006). This is consistent with previous research on reactance that has focused on behavioral intentions in hypothetical scenarios (Sittenthaler et al., 2015) or behavior in nonsocial settings (Chartrand et al., 2007). Our study complements and extends this research by providing evidence of the motivation to act against the restriction of one's freedom of choice during social decisions with actual consequences. A direct comparison of the predictors based on the self-report data revealed that a combination of the social cognitions perceived distrust and understanding explained individual control-averse behavior best at the behavioral level.

At the neural level, we found that control-averse behavior could be predicted by functional connectivity between the right IPL and the bilateral dlPFC/middle frontal gyrus. Our finding is specific to the right IPL, which corroborates previous work examining its role in subjective choice restrictions (Filevich et al., 2013). The involvement of both IPL and dlPFC in control-averse behavior could be attributed to their functions suggested in previous neuroimaging studies. The IPL has traditionally been associated with the reorienting of attention to both social and nonsocial stimuli (Corbetta et al., 2008), as well as number processing (Dehaene et al., 2003). In addition, more recent work has linked the IPL to social distance encoding (Chiao et al., 2009; Parkinson et al., 2014), suggesting that the IPL might perform analogous operations in visuospatial and social contexts (Yamazaki et al., 2009; Parkinson et al., 2014). Therefore, it seems plausible that the differential IPL activation during decisions in the controlled compared with the free condition might reflect the encoding of or attention reorientation to the context (i.e., being controlled or not) that is relevant for the decision (i.e., to counteract or not). The differential IPL activation alone, however, did not explain individual differences in control-averse behavior, suggesting that the IPL encodes the difference between the controlled and the free condition regardless of the subjects' individual control aversion. Instead, individual differences in control-averse behavior could be explained by the connectivity of right IPL with the dlPFC, two regions that are connected directly through fiber tracts (Mars et al., 2012). Moreover, the IPL and regions in the lateral PFC show robust intrinsic functional coupling (Mars et al., 2011) and increased task-based coupling during changes of choice strategy (Daw et al., 2006; Boorman et al., 2009). Follow-up studies should investigate whether individual differences in anatomical or resting-state functional connectivity between the IPL and dlPFC might contribute to control-averse behavior.

The dlPFC has commonly been associated with cognitive control (MacDonald et al., 2000; Miller and Cohen, 2001) and overcoming conflicts in decisions that require self-control (Knoch et al., 2006; Hare et al., 2009; Figner et al., 2010; Baumgartner et al., 2011). Correspondingly, the notion that control-averse behavior requires cognitive control is supported by our behavioral data: Although all subjects demonstrated an intrinsic motivation to choose a high level, control-averse subjects were more likely to dislike the restriction of their freedom of choice and to feel the urge to use their remaining freedom of choice. This suggests that control-averse subjects perceived the decisions in the controlled condition as a conflict between the general motivation to choose a high level and the condition-specific motivation to act against the restriction. Given its suggested role in cognitive control, this could explain why the dlPFC was more strongly recruited by control-averse subjects during decisions in the controlled condition, as indicated by the connectivity analysis and illustrated in the time course plots.

Furthermore, model comparisons indicate that the right IPL–dlPFC connectivity explains additional variance of the individual control-averse behavior that has remained unexplained by self-reports alone. More specifically, we find that the neural data complement the self-reports of social cognitions. Together, these two predictors explain a sizable amount of variance in the control-averse behavior and provide the best data fit among the tested models. The IPL cluster that we find lies in close proximity to the temporoparietal junction (Mars et al., 2012; Igelström et al., 2015), which is considered a key region in social cognition (Decety and Lamm, 2007; Cabeza et al., 2012; Carter and Huettel, 2013; Krall et al., 2015). It has been proposed that the IPL shares information with the temporoparietal junction via joint connections in the dlPFC/middle frontal gyrus (Corbetta et al., 2008), matching the target region of our connectivity analysis. Consistent with this notion, we found that the right IPL–dlPFC connectivity partially mediates the association between social cognition and control-averse behavior. The partial mediation and model comparisons further suggest that the right IPL–dlPFC connectivity explains variance that could not be captured by self-reports. This emphasizes once more that, for a comprehensive understanding of a complex human behavior such as control-averse behavior, it is essential to incorporate neurophysiological factors. Although the IPL and dlPFC certainly have intricate roles in decision making, together, our data provide evidence that the controlled condition represents a socially salient event and that the right IPL–dlPFC connectivity might contribute to the integration of social cognition into control-averse behavior.

Last, it is important to acknowledge the limitations of our study and provide suggestions on how to address them in future work. First, it would be interesting to see whether our results generalize to nonsocial scenarios. Falk and Kosfeld (2006) have demonstrated, however, that replacing player A with a computer algorithm eliminates control-averse behavior, suggesting that the aversion to the choice restriction might be confounded with the social aspect in our task. Therefore, designing a study that analogously varies the degree of choice restrictions in both a social and nonsocial context could be an interesting future endeavor.

Furthermore, we opted for a small number of trials to increase credibility and to limit possible habituation and attention biases. This means that, whereas our neuroimaging results survive whole-brain correction, some brain activation might have gone undetected. Using a greater number of trials, however, would have come at the risk of a less robust measure of control-averse behavior. In the current data, the robustness of our measure of control-averse behavior is supported by the consistent correlations with the affect and self-report ratings. Similar sanity checks should be incorporated in future neuroimaging studies on control-averse behavior.

In conclusion, this study provides first insights into the neural drivers of individual differences in control-averse behavior, a social phenomenon that is ubiquitous in our society. The prevalence of control-averse behavior and its potential negative consequences have become evident in previous behavioral studies. Advancing our understanding of the mechanisms that give rise to individual differences in control-averse behavior therefore represents an important research goal. Here, we have approached this goal by identifying a neural mechanism that can explain individual differences in control-averse behavior. Our results suggest that a key driver of control-averse behavior is the connectivity between brain regions that are reliably, albeit not exclusively, involved in attention reorientation and cognitive control. This connectivity complements what could be measured by self-reports alone and thereby improves our understanding of the mechanisms underlying control-averse behavior. Although more work is needed to investigate the exact neural computations and extend these findings to more complex social interactions, this study has brought us a significant step forward in unraveling the drivers of individual differences in control-averse behavior.
